# Factors Associated With Adult Incarceration Among People With Opioid Use Disorder in New South Wales, Australia

**DOI:** 10.1111/dar.70153

**Published:** 2026-04-14

**Authors:** Christel Macdonald, Chrianna Bharat, Louisa Degenhardt, Matthew Hickman, Jack Stone, Rachel Sutherland, Mary Harrod, Jason Grebely, Thomas Santo

**Affiliations:** ^1^ National Drug and Alcohol Research Centre UNSW Sydney Sydney Australia; ^2^ School of Population and Global Health University of Western Australia Perth Australia; ^3^ Population Health Sciences, Bristol Medical School University of Bristol Bristol UK; ^4^ NSW Users and AIDS Association Sydney Australia; ^5^ The Kirby Institute UNSW Sydney Sydney Australia

**Keywords:** discrete‐time event analysis, incarceration, injecting drug use, offending, opioid use disorder, social determinants of justice

## Abstract

**Introduction:**

People with opioid use disorder (OUD) are at elevated risk of incarceration. Understanding factors linked to this risk may support more targeted prevention and intervention efforts. This study used cross‐sectional survey data to examine associations with time to first incarceration and repeated incarceration among individuals with OUD. Additionally, we wanted to examine the characteristics of people with opioid dependence who had been incarcerated.

**Methods:**

Data were drawn from 297 adults with OUD in New South Wales, Australia (2023–2024). Participants completed structured interviews covering socio‐demographics, substance use, adverse childhood experiences and criminal justice contact. A discrete‐time event analysis examined correlates of first adult incarceration from age 18.

**Results:**

Overall, 58% of participants reported having been incarcerated. In adjusted models, injecting drug use increased the risk of first‐time incarceration in any given year (aOR: 3.55, 1.08–11.66), as did childhood exposure to household substance use (aOR: 1.80, 1.15–2.82), and being male (aOR: 1.83, 1.24–2.69). Greater secondary education reduced incarceration odds (aOR: 0.56, 0.37–0.86). In any given year, younger adults (18–24 years) were at highest risk of incarceration, whereas those aged 35 or older had notably lower odds (aOR: 0.16, 0.09–0.29).

**Discussion and Conclusions:**

Injecting drug use, limited education, and childhood exposure to household substance use were associated with higher incarceration risk. There is a need to consider broader social and developmental factors in supporting individuals with OUD. While systemic change is complex, this study adds to the evidence base that can inform more integrated approaches to reducing incarceration risk.

## Key Point Summary

- Time‐to‐event analysis examined predictors of incarceration in opioid‐dependent adults.
- The strongest predictor of incarceration in any given year was injecting drug use.
- Adverse childhood experiences, gender, and education also were strongly associated with incarceration.

## 1 Introduction

Opioid use disorder (OUD) is a major public health issue with multiple causes of premature mortality and morbidity including overdose, HIV, hepatitis C virus (HCV) infection and transmission, and bacterial endocarditis [1, 2]. There are also broader social costs such as reduced employment and increased risk and costs of crime [1] and incarceration. Rates of incarceration for people with substance use dependence are high globally [3] and in Australia [4], and are associated with increased health‐related harms, including higher prevalences of long‐term health conditions and mental disorders compared to the general population [5]. The negative impacts of incarceration continue after release, with individuals released from prison having higher hospital admission and mortality rates than the general population, lower rates of employment, and lack of housing [5]. Additionally, Australian studies have shown high rates of injury [6], emergency department presentations [7] and resumption of injecting drug use following release from prison [8].

In addition to the general risks of incarceration, people with OUD who are incarcerated are vulnerable to specific risks like exposure to infectious diseases through sharing needles and syringes to inject drugs [9, 10]. Among people who inject drugs (PWID), needle sharing is particularly elevated during periods of incarceration due to lack of access to sterile equipment [11, 12]. Additionally, the period following release from prison is also associated with increased risk of acquiring HIV and hepatitis C among PWID [13]. Similarly, studies have demonstrated that individuals released from prison have a significantly elevated risk of death than the general population [14, 15], and this is largely driven by drug‐related deaths in the first 4 weeks after release [16]. Moreover, opioids have been implicated in a quarter of all post‐release deaths and more than half of all overdose deaths of those released from prison [14, 17, 18].

Given these pervasive harms, exploring the social and developmental factors associated with incarceration among people with OUD is important for understanding broader determinants that may inform future intervention efforts. The World Health Organization defines social determinants of health (SDH) as the conditions in which people are born, grow, work, live and age, and the broader systems that shape their daily lives [19]. These social determinants that shape health outcomes are also posited to drive interactions with the justice system and contribute to disparities in justice outcomes. For example, individuals with socioeconomic disadvantage, those experiencing drug and alcohol use disorders and people with mental disorders are among the populations with elevated rates of incarceration and contact with police [20]. In their social determinants of justice model, McCausland and Baldry argue that social and economic issues such as substance use, unemployment and social isolation, and early life experiences like family violence and child abuse and/or neglect are some of the factors contributing to future criminalisation and incarceration [20]. These factors are referred to as social determinants of justice [20].

There have been some empirical studies examining potential social determinants of justice, such as substance use and adverse childhood experiences. These studies show that substance use disorders significantly increase the odds of incarceration, with mixed findings on the impact of age of onset of substance use [21, 22, 23]. Additionally, there is some evidence that a history of child abuse and parental incarceration increases the odds of being incarcerated at younger ages [21], while factors like age, gender and education impact lifetime incarceration odds among individuals with opioid dependence [24]. These studies, however, are limited by the fact they either did not focus on individuals with opioid dependence [21], only examined incarceration into early adulthood [22], or did not consider a broad range of factors that may impact incarceration [23]. Larney et al. examined a broader range of factors among the population of interest on lifetime incarceration [24]. The present study seeks to expand on the findings of Larney et al. by examining what factors are significantly associated with first‐time adult incarceration.

The current study aimed to address some of these limitations by conducting a time‐to‐event analysis, which enabled an examination of the risk in any given year of becoming incarcerated from 18 years of age, and the time‐varying factors that increase or decrease this risk. Specifically, the aims of the current study were to examine the age of first adult incarceration and the time to this event, and to identify the factors associated with time to incarceration in a sample of individuals with opioid dependence. Additionally, we explored the factors associated with repeat incarceration.

## 2 Methods

### 2.1 Design and Setting

The Opioid Dependence Survey was conducted in New South Wales (NSW) and was a cross‐sectional survey of individuals living with opioid dependence in NSW. Participants took part in an interview either face‐to‐face, online or over the phone between October 2023 and March 2024. Participants were recruited through flyers displayed at a variety of sites and distributed or advertised through multiple forms of communication including word‐of‐mouth. Locations included private and public opioid agonist treatment (OAT) clinics, needle and syringe program sites, pharmacies, community service centres, health clinics and other locations that provide drug and alcohol services. Peer‐based organisations such as NSW Users and AIDS Association assisted in devising strategies to distribute the flyers to members through physical communication and/or online outlets. Interviews took approximately 1 hour. Participants received AUD$40 as reimbursement for their time. Ethical approval was sought and obtained from the UNSW Human Research Ethics Committee and from St Vincent's Hospital Human Research Ethics Committee Sydney (2023/ETH01404). For further details on the survey and full sample, see Santo et al. [25].

### 2.2 Study Sample

To be eligible, participants had to be 18 years of age or older and either currently on OAT or dependent on non‐prescribed pharmaceutical opioids and/or heroin (use on at least 21 of the past 28 days). Individuals who were unable or unwilling to give consent were ineligible. As the outcome of interest was adult incarceration, those who reported their first age of incarceration before the age of 18 were excluded from analysis. This is because the survey only asked, ‘at what age were you first incarcerated?’ and did not have separate questions for juvenile incarceration and adult incarceration. Thus, if, for example, an individual reported their first incarceration at age 14, there was no information about the age of their first adult incarceration. Incarceration refers to prison or juvenile detention, in which an individual was detained in custody, including both sentenced and remand.

### 2.3 Measures

#### 2.3.1 Outcome Variable

The primary outcome of interest was age at first incarceration (at age of 18 years or over). This was obtained from the question in the survey which asked individuals the age of their first incarceration. Additional variables of interest related to incarceration were whether individuals had last been in prison in the past 12 months or more than 12 months ago, how many times they had been to prison, the duration of their most recent incarceration, and the reason for their last incarceration.

#### 2.3.2 Covariates

Key demographic details included age, gender, education history, and employment. Participants were also asked about their substance use history, opioid dependence, and OAT history, mental health, adverse childhood experiences, trauma, and incarceration and arrest. Participants were also asked if they had ever been arrested for opioid use or possession. Current mental health was measured using the Patient Health Questionnaire‐9, the Suicidal Ideation Attributes Scale, the Generalized Anxiety Disorder (GAD‐7) scale and the Primary Care PTSD Screen for DSM‐5 (PC‐PTSD‐5). The Adverse Childhood Experiences Questionnaire (ACE‐Q) [26] was used to assess childhood experiences and trauma. We examined the individual ACE items rather than a total ACE score because past research indicates specific forms of adversity are more predictive of criminal‐legal outcomes, and item‐level measurement explains substantially more variance than summed scores [27, 28].

Covariates for the time‐to‐event analysis were focused on social determinants of justice, and were included on the basis that, either on past research or on a priori grounds, they may be expected to influence adult incarceration. These were age category, gender, highest education attainment [24], alcohol use [21, 22, 24], adverse childhood experiences and trauma [21, 24], and age at first illicit drug use [21, 22, 23, 24]. Age of first illicit drug use was modelled as a time‐dependent indicator variable. Although data were collected cross‐sectionally, we retrospectively constructed this variable to reflect the timing of onset. Age of first illicit drug use was categorised into one variable with three levels: no drug use, drug use but no injecting and injecting drug use. This variable was derived from questions in the survey that asked participants at what age they first used an illicit substance and what age they first injected a drug. This allowed us to separate out the effect of drug use with no injecting from injecting drug use and represents a marker of increasingly frequent or serious drug use as age of onset of diagnosis was unknown.

#### 2.3.3 Data Analysis

Descriptive statistics were conducted and bivariate analyses used to examine difference between: (i) individuals who had never been incarcerated; (ii) individuals who had been incarcerated within the last 12 months; and (iii) individuals whose most recent incarceration was longer than 12 months ago. We examined these groups because incarceration is associated with substantial instability in housing, employment and other social circumstances, and these factors might shift over time and may differ meaningfully by recency of incarceration. Stratifying by recency of incarceration may therefore provide a clearer description of demographic variation within this population. We also compared characteristics of participants first incarcerated as juveniles (< 18 years) versus those first incarcerated as adults (≥ 18 years) (see Table S1). For descriptive comparisons between groups, *p* values were determined using Pearson's chi‐squared test. For non‐normally distributed data, Wilcoxon rank‐sum tests of significance are reported. A cumulative incidence plot was generated using Kaplan–Meier methods to estimate the proportion of participants experiencing first adult incarceration by age. The analysis was conducted in R (v4.4.1) using the survival [29] and survminer [30] packages, with censoring applied at age of interview or first incarceration.

Incarceration profiles of people who had been incarcerated in the last 12 months versus those who had been incarcerated more than 12 months ago were identified, examining: mean age of first incarceration, number of times incarcerated, offence type for last incarceration and whether the participant had ever been arrested for opioid use or possession.

The primary outcome being modelled was first adult (18 years or older) incarceration. Time‐to‐event was calculated from 18 years of age. Univariate and multivariate discrete‐time logistic regression models were used to examine predictors of first adult incarceration. These analyses were conducted using person‐year as the unit of analysis and a logistic link function. A person‐year dataset was created in which each year in the life of each respondent during which they were at risk of being incarcerated, from the age of 18 up to the age of incarceration or age at interview (whichever came first), was treated as a separate observational record. The year of incarceration was coded 1 and earlier years coded 0. Survival coefficients and standard errors are presented as odds ratios and 95% confidence intervals (CI). After running the univariate analyses, anything with a *p* value ≤ 0.1 was entered into the multivariate model. This threshold balanced inclusion of moderately associated predictors with model parsimony, while the events‐per‐variable ratio (173 events/8 predictors) exceeded recommended thresholds for stable estimation [31, 32]. All analyses were conducted using STATA 18, with a significance level of 0.05.

To assess the robustness of findings, two additional negative binomial regression models were conducted to examine predictors of repeated incarceration. The first included the full sample by adding participants whose first incarceration occurred before age 18 (*n* = 82), to assess the impact of including juvenile‐onset cases. The second was a zero‐truncated model restricted to individuals who had been incarcerated at least once, to explore factors associated with the number of incarcerations among this subgroup. See Appendix A for further methodological details. These models used the same covariates as the primary analysis and are presented in Appendix Tables B2 and B3.

## 3 Results

### 3.1 Descriptive Statistics

A total of 405 participants were recruited to the study. Eighty‐two individuals who reported their first age of incarceration before the age of 18, and 26 people with missing information regarding age of first incarceration were excluded from analysis. This resulted in a final sample of 297 participants. Table 1 shows the descriptive statistics of the sample. Men comprised the majority of the sample (65%) and the mean age was 44 years. Eighty‐four percent of the sample had experienced homelessness in their lifetime and 84% were unemployed at the time of interview. Additionally, the median age of first injecting drug use was 18 years (interquartile range 16–23) for the whole sample. The median age of first opioid use was similar (19 years; interquartile range 16–24).

**TABLE 1:** Sample statistics stratified by incarceration history.

	Total sample (%)	Incarcerated more than 12 months ago (%) (*A*)	Incarcerated in past 12 months (%) (*B*)	Never incarcerated (%) (*C*)	*p* (*A* vs. *B*)	*p* (*A* vs. *C*)	*p* (*A* + *B* vs. *C*)
*N*	297	125	48	124			
Mean age, years (SD)	44.72 (9.88)	46.94 (9.42)	41.69 (8.33)	43.65 (10.45)	< 0.001	0.245	0.116
Gender					0.039	0.391	0.054
Male	192 (65)	82 (66)	40 (83)	70 (56)			
Female	101 (34)	42 (33)	7 (14)	52 (42)			
Highest level of education					0.002	0.061	0.001
Less than 10 years secondary education	76 (25)	32 (34)	24 (50)	20 (16)			
10 years of secondary education or higher	220 (74)	92 (66)	24 (50)	104 (84)			
Employment status					0.072	0.600	0.206
Employed	48 (16)	21 (17)	3 (6)	24 (19)			
Unemployed	249 (84)	104 (83)	45 (94)	100 (81)			
Ever experienced homelessness	251 (84)	111 (89)	47 (98)	93 (75)	0.056	0.005	< 0.001
Ever injected drugs	276 (93)	119 (95)	48 (100)	109 (88)	0.122	0.038	0.004
Hazardous drinking on AUDIT‐C^*1*^	124 (42)	46 (37)	18 (37)	60 (48)	0.932	0.064	0.050
Ever been on OAT	269 (90)	116 (93)	43 (89)	110 (88)	0.487	0.265	0.352
Currently on OAT	230 (85)	100 (85)	33 (77)	97 (88)	0.191	0.547	0.250
Arrested for opioid use/possession	125 (43)	57 (46)	28 (61)	40 (32)	0.093	0.023	0.002
Adverse childhood experiences							
Household member used substances	152 (59)	73 (68)	31 (77)	48 (44)	0.271	< 0.001	< 0.001
Mother treated violently	96 (38)	40 (38)	24 (60)	32 (29)	0.018	0.176	0.016
Physical neglect	68 (27)	31 (29)	14 (35)	23 (21)	0.502	0.169	0.082
Physical abuse	125 (49)	49 (46)	24 (58)	52 (47)	0.165	0.778	0.798
Emotional neglect	127 (50)	55 (52)	16 (39)	56 (52)	0.162	0.996	0.575
Emotional abuse	146 (57)	60 (56)	25 (61)	61 (56)	0.589	0.987	0.814
Sexual abuse	106 (41)	41 (38)	21 (50)	44 (40)	0.193	0.758	0.841
Household member had a mental illness	102 (40)	44 (42)	19 (47)	39 (36)	0.544	0.386	0.239
Parent went to prison	68 (27)	28 (27)	22 (55)	18 (16)	0.001	0.071	0.001
Parents separated or divorced	149 (59)	67 (64)	23 (57)	59 (54)	0.484	0.150	0.203

Abbreviations: AUDIT, Alcohol Use Disorders Identification Test; OAT, opioid agonist treatment.

More than half (58%) had previously been incarcerated. The incarceration profiles for individuals who had ever been incarcerated are shown in Table 2. There were no statistically significant differences between those last incarcerated within the past 12 months and those incarcerated more than 12 months ago in the mean number of times incarcerated (4.5 vs. 5.4; *p* = 0.085) or mean age at first adult incarceration (24.7 vs. 24.6 years; *p* = 0.795). These analyses were exploratory, and given the modest sample size, statistical power to detect small differences was limited. Among those who had ever been incarcerated (including those first going to prison as juveniles), the mean age of first incarceration was 14.4 years (SD = 2.0) for individuals first incarcerated as juveniles and 24.6 (SD 7.8) for those first incarcerated as adults (see Appendix Table B1 for additional data).

**TABLE 2:** Incarceration profiles of people who have been incarcerated ever and incarcerated in last 12 months.

Variable	Total incarcerated sample, *n* (%)	Incarcerated more than 12 months ago, *n* (%)	Incarcerated in past 12 months, *n* (%)	*p* (12 months vs. lifetime)
*N*	173	125	48	
Number of times incarcerated, mean (SD)	4.7 (4.8)	4.5 (4.8)	5.4 (4.9)	0.085^a^
Age of first adult incarceration, mean (SD)	24.6 (7.8)	24.7 (7.7)	24.6 (8.0)	0.795^a^
*Offence type for last incarceration* ^*b*^				
Assault	42 (25)	28 (23)	14 (31)	0.017^c^
Robbery/extortion	40 (24)	30 (25)	10 (22)	
Fraud/deception	10 (6)	7 (6)	3 (7)	
Homicide	2 (1)	1 (1)	1 (2)	
Drug and drug related offences	36 (22)	34 (28)	2 (4)	
Other	36 (22)	21 (17)	15 (33)	

^a^ Wilcoxon rank‐sum test statistic.

^b^ Missing for 7 people.

^c^ Chi‐square statistic.

### 3.2 Time to First Adult Incarceration

**FIGURE 1:**
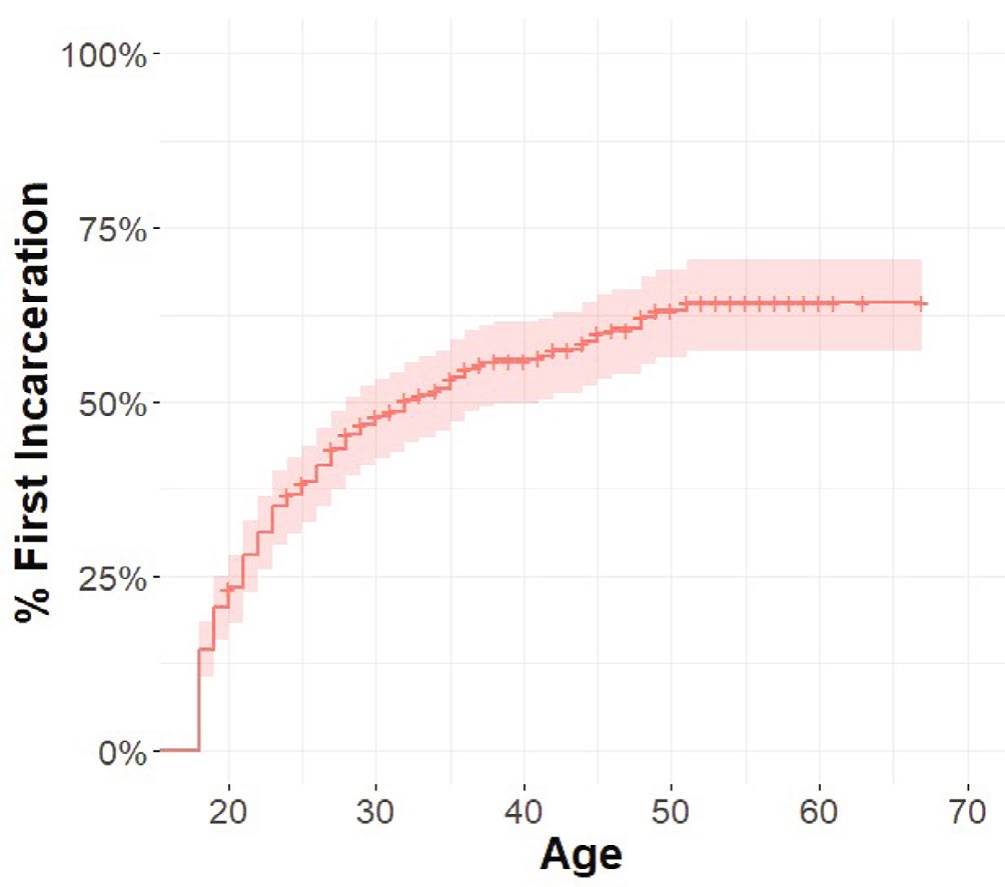
Cumulative proportion (1‐survival) of participants experiencing their first adult incarceration among people who use extra‐medical opioids in NSW (*N* = 297). Person‐years begin at 18 years; Participants censored (marked a ‘+’) at age of interview if they have not been incarcerated by that point. Shaded areas represent 95% confidence intervals. This curve reflects the cumulative incidence (i.e., a ‘failure curve’ or ‘1‐survival’) for first adult incarceration.

The cumulative proportion of participants incarcerated by age is shown in Figure 1. The plot shows that with increasing age, the proportion of people ever incarcerated increases, with the sharpest increase occurring in the first 10 years of adulthood (ages 18–28); 45% of participants had been incarcerated by age 28.

Table 3 shows the results of the regression of the discrete‐time survival analysis. Men had 1.83 (95% CI 1.24, 2.69) times the odds of being incarcerated than women in any given year. Individuals who had injected drugs had 3.65 (95% CI 1.12, 11.91) times the odds of being incarcerated in any given year than those who had no history of injecting by that year. Individuals whose household member had used psychoactive substances in their childhood had twice the odds of being incarcerated in any given year (adjusted odds ratio [aOR] 2.07; 95% CI 1.36, 3.16). Having completed education past year 10 was a protective factor, nearly halving the odds of being incarcerated in any given year (aOR 0.56; 95% CI 0.37–0.86). There was a reduced risk of incarceration with increasing age. As age increased, the odds of being incarcerated reduced; compared to the younger age group (18–24 years), this effect was strongest in the 30–34‐year (aOR 0.35; 95% CI 0.19, 0.63) and 35 and older age groups (aOR 0.16; 95% CI 0.09, 0.29). In the negative binomial regressions that included people who had been first incarcerated before adulthood, in both models, being male and completing fewer than 10 years of schooling were consistently associated with higher rates of repeated incarceration, while older age at first opioid use was protective; physical neglect was associated with higher rates of repeated incarceration in the full sample (Tables B2 and B3, Appendix).

**TABLE 3:** Factors associated with adult incarceration.

Incarceration	Unadjusted OR (95% CI)	*p*	Adjusted OR (95% CI)	*p*
Age category				
18–24 (ref)	—	—	—	—
25–29	0.49 (0.32, 0.74)	0.001	0.52 (0.33, 0.82)	0.005
30–34	0.28 (0.15, 0.50)	< 0.001	0.33 (0.18, 0.61)	< 0.001
35+	0.21 (0.13, 0.33)	< 0.001	0.15 (0.09, 0.28)	< 0.001
Male	1.59 (1.13, 2.22)	0.007	1.86 (1.27, 2.71)	0.001
Education > yr. 10	0.49 (0.35, 0.69)	< 0.001	0.62 (0.41, 0.94)	0.024
No drug use (ref)	—	—	—	—
Non‐injecting drug use (time‐dependent)	1.40 (0.53, 3.69)	0.490	1.98 (0.58, 6.74)	0.272
Injecting drug use (time‐dependent)	3.02 (1.20, 7.65)	0.019	3.65 (1.12, 11.91)	0.032
Alcohol use	1.94 (0.84, 4.45)	0.118	—	—
Adverse childhood experiences				
Household member used substances	2.21 (1.53, 3.18)	< 0.001	2.07 (1.36, 3.16)	0.001
Mother treated violently	1.51 (1.08, 2.13)	0.017	0.91 (0.61, 1.46)	0.625
Physical neglect	1.39 (0.96, 2.00)	0.076	1.20 (0.81, 1.79)	0.361
Physical abuse	1.02 (0.73, 1.42)	0.912	—	—
Emotional abuse	1.00 (0.71, 1.40)	0.995	—	—
Emotional neglect	0.92 (0.66, 1.29)	0.628	—	—
Sexual abuse	1.02 (0.73, 1.43)	0.891	—	—
Household member had a mental illness	1.29 (0.92, 1.82)	0.139	—	—
Parent went to prison	1.94 (1.35, 2.77)	< 0.001	1.45 (0.97, 2.16)	0.068
Parents separated or divorced	1.27 (0.90, 1.79)	0.178	—	—
Constant	—	—	0.01	< 0.001

## 4 Discussion

### 4.1 Summary and Interpretation of Main Findings

In this sample of individuals with opioid dependence, more than half had been incarcerated at least once—often multiple times—and nearly half had been arrested for opioid use or possession. Examining risk across adulthood, we found that injecting drug use was the strongest predictor of first incarceration in any given year, followed by being male, having a household member who used substances in childhood, younger age, and lack of secondary education. Additional analyses among those first incarcerated as juveniles reinforced these patterns: being male and having less than 10 years of schooling were consistently associated with repeated incarceration, older age at first opioid use was protective, and physical neglect emerged as an additional risk factor in some models. These findings underscore how social disadvantage, early‐life adversity, and injecting patterns may contribute to earlier onset of incarceration among people with lived experience of opioid dependence, highlighting the potential benefits of strategies that address these cumulative vulnerabilities as well as preventive interventions targeting adolescents and young adults.

The findings regarding injecting drug use are consistent with the findings of a global systematic review, which found high rates (58%) of lifetime incarceration among people who inject drugs [33]. Importantly, the current study adds to the literature by demonstrating that in any given year, injecting drug use increases the risk of first‐time incarceration. Longitudinal studies using national survey data have found childhood physical and sexual abuse increased the odds of earlier incarceration onset [21]. Surveys focusing on individuals with opioid dependence, however, have not found an impact of child abuse and neglect on risk of incarceration [24]. The current study aligns with these latter findings. Important differences between the present study and past research that has found an effect of physical abuse may explain these different findings. The rate of any child abuse among the nationally representative sample analysed in Barnert et al. [21] was 20%. In contrast, in our cohort, rates of physical abuse were high, and were similar across those who had (47%) and had not been (42%) incarcerated. Sexual abuse was similarly high for both groups (40% for never incarcerated, 36% for those who had been). Although Larney et al. [24] did not give a detailed breakdown of subtypes of abuse and neglect by incarceration, their sample also demonstrated very high rates of at least one type of childhood maltreatment. Thus, it appears that adverse childhood events may be commonly shared experiences among individuals with OUD and hence do not pose an additional risk for adult incarceration among this group. Rather, other factors like the age of onset of injecting drug use appear to be driving forces for adult incarceration among people with OUD.

Importantly, the time‐to‐event analysis demonstrates that in any given year when an individual is aged 18–24, their odds of first‐time incarceration are significantly higher than individuals not in this age group. It is probable that this is because the ‘riskiest’ adults, or those with the strongest risk factors for incarceration, are likely to be incarcerated at younger ages. Evidence shows that criminal behaviour begins to rise in early adolescence and peaks between the ages of 18–24 before declining in early adulthood [34], and younger offenders tend to be treated more punitively than older offenders [35]. Additionally, in 2025, Australians aged 18–24 had a higher imprisonment rate than those aged 35 years and over, with rates peaking in the late 20s to early 30s [36]. The findings of the current study reinforce the importance of early intervention and prevention strategies targeting young adults, who are at the greatest risk of entering the criminal justice system for the first time.

We found that non‐injecting drug use did not significantly impact the risk of adult incarceration in both unadjusted and adjusted models. It is important to remember that in our analysis, the time‐to‐event was calculated from age 18. In our cohort, the overwhelming majority of people (83%) had tried non‐injecting illicit drugs before the age of 18. In contrast, only 39% of the cohort had tried injecting drugs before the age of 18. Thus, there was likely not enough variability on age of first non‐injecting drug use in the sample to identify an impact on adult incarceration. Additionally, the finding that non‐injecting drug use was not associated with incarceration aligns with the broader literature findings that incarceration and reincarceration are concentrated among PWID [37, 38, 39] and that the prevalence of injecting drug use within prison populations is much higher than in the community [40]. While we found an initial impact of parental incarceration during childhood, this effect was no longer significant once other factors in the model were accounted for. A stepwise regression showed that parental substance use and education reduced the coefficient and significance of parental prison; however, only age category made parental incarceration non‐significant.

### 4.2 Implications

The findings of the present study suggest a potential association between a number of factors and adult incarceration. Firstly, schooling appears to be a protective factor, with the odds of incarceration approximately half that of those who had completed less than 10 years of school education. Secondly, the findings regarding the 18–24‐year age period and children exposed to parental substance use suggest two potential target groups for early intervention. While further research is needed to determine causality, the results of the current study suggest that interventions improving re‐engagement with education, intervening early in young adults, and preventative efforts focusing on family support may be impactful in reducing the likelihood of future incarceration.

Lack of stable housing and unemployment in the sample—factors which prior research suggests are associated with incarceration [20, 41]—suggest that interventions must be comprehensive and multifaceted, and address more than just substance use. There are a number of programs designed to re‐engage young people with employment and education, including mentoring, school‐based learning and work placements [42]. Reviews of the evidence base, however, indicate that the demonstrated effectiveness of programs is limited [42, 43, 44]. Some support exists for intensive multi‐component interventions in reducing unemployment [44]. To minimise the harms associated with incarceration, diversionary schemes that refer defendants into treatment should be prioritised, particularly since prison has been consistently shown to not encourage deterrence [45]. Studies evaluating drug courts, which divert individuals with drug dependence who are charged with offences away from incarceration, have found they significantly reduce arrests, drug charges and recidivism [46, 47]. Additionally, other diversionary schemes that refer individuals into treatment at earlier stages of a criminal offence, for example upon first contact with police, have been shown to reduce the risk of reoffending and reincarceration, as well as deaths over a two‐year follow‐up period [45]. This is particularly relevant to the current study, given we found that a significantly higher proportion of incarcerated individuals had been arrested for opioid use or possession compared to those who had never been incarcerated. The point of arrest indicates an important timepoint of early intervention via diversionary programs [45].

### 4.3 Limitations

The present study has some limitations that need to be discussed. Firstly, given the survey was cross‐sectional, we cannot infer causal associations between the factors examined and incarceration. Importantly, however, by conducting a time‐to‐event analysis, only variables that occurred prior to 18 years of age or first adult incarceration were included. Thus, the possibility that incarceration increased the risk, for example, of injecting drug use can be eliminated. There are, however, likely confounders for incarceration that co‐occur with early substance use that we were unable to measure, such as delinquency. Secondly, although the sample was recruited from many locations using diverse methods across the state, the cohort may not be a representative sample of people with opioid use disorder, particularly of those living in regional or remote areas. Thirdly, the age of first drug use and age of first incarceration were recalled retrospectively and thus are subject to recall bias and may not be accurate. Finally, because mortality is high among people recently released from prison, our cross‐sectional sample may underrepresent individuals with the highest risk profiles, introducing potential survivor bias.

## 5 Conclusions

Few studies have examined risk factors for incarceration among people who use opioids. The findings from our time‐to‐event analysis provide further evidence for factors contributing to adult incarceration among this population. Notably, injecting drug use, exposure to substance use in the home during childhood, and limited secondary education were all associated with an increased risk of first incarceration in any given year. The 18–24‐year age bracket represented the riskiest time period for first‐time adult incarceration in any given year.

The findings of the current study underscore the importance of early intervention and prevention strategies, particularly targeting young adults who are at the greatest risk of entering the criminal justice system for the first time. Future research is needed to establish causality between these factors and incarceration in a larger sample size. Additionally, future studies may examine the impact of interventions that address injecting drug use, adverse childhood experiences, and education retention and re‐engagement initiatives among people with opioid dependence.

## Author Contributions

**Christel Macdonald:** conceptualisation, data curation, methodology, formal analysis, writing – original draft and review and editing. **Chrianna Bharat:** conceptualisation, methodology, supervision, writing – original draft and review and editing. **Louisa Degenhardt:** conceptualisation, methodology, writing – original draft and review and editing, supervision. **Matthew Hickman:** methodology, writing – review and editing. **Jack Stone:** methodology, writing – review and editing. **Rachel Sutherland:** methodology, writing – review and editing. **Mary Harrod:** methodology, writing – review and editing. **Jason Grebely:** writing – review and editing. **Thomas Santo Jr:** conceptualisation, methodology, supervision, writing – original draft and review and editing.

## Funding

This study was supported by an Australian National Health and Medical Research Council (NHMRC) Program grant (1150078), an NHMRC Investigator Grant (1176131, 2016825, 2034002) and an NHMRC Senior Principal Research Fellowship (1135991) to LD; National Institute on Drug Abuse (1R01DA059822‐01A1); and the National Drug and Alcohol Research Centre, UNSW Sydney. The National Drug and Alcohol Research Centre is funded by the Australian Government Department of Health. The views expressed in this publication do not necessarily represent the position of the Australian Government.

## Disclosure

This research was produced in whole or part by UNSW Sydney researchers and is subject to the UNSW Intellectual property policy. For the purposes of Open Access, the author has applied a Creative Commons Attribution CC BY licence to any Author Accepted Manuscript (AAM) version arising from this submission.

## Conflicts of Interest

Past 3 years, J.G. is a consultant or advisor and has received research grants from Abbvie, Abbott, bioLytical, Cepheid, Gilead Sciences, Hologic, and Roche. All other authors declare no conflicts of interest.

## Supporting information


**Appendix A** Supplementary methods.
**Appendix B:** Supplementary results.
**Table B1:** Comparison of characteristics between people who were first incarcerated as juveniles and people first incarcerated as adults.
**Table B2:** Negative binomial regression for whole sample (*N* = 357).
**Table B3:** Negative binomial regression—zero‐truncated model (*N* = 233).
**Table B4:** Descriptive statistics of number of times incarcerated.
**Table B5:** Multivariate regression using ACE as a count score.

## Data Availability

The data that support the findings of this study are available on request from the corresponding author. The data are not publicly available due to privacy or ethical restrictions.
