# Supplementary material for: Factors Associated With Adult Incarceration Among People With Opioid Use Disorder in New South Wales, Australia
Source: Drug Alcohol Rev. 2026 Apr 14;45(4):e70153. doi: 10.1111/dar.70153 (PMC13078918; doi:10.1111/dar.70153)
Supplement: Supplementary file 1 — Appendix A Supplementary methods. Appendix B: Supplementary results. Table B1: Comparison of characteristics between people who were first incarcerated as juveniles and people first incarcerated as adults. Table B2: Negative binomial regression for whole sample (N = 357). Table B3: Negative binomial regression—zero‐truncated model (N = 233). Table B4: Descriptive statistics of number of times incarcerated. Table B5: Multivariate regression using ACE as a count score. [file DAR-45-0-s001.docx]

**Web appendices for:**

**Factors associated with adult incarceration among people with opioid use disorder in New South Wales, Australia**

**Appendix A: Supplementary methods**

**Additional descriptive comparisons**
We compared characteristics of participants first incarcerated as juveniles (<18 years) versus those first incarcerated as adults (≥18 years). These comparisons are reported in Table B1.

**Sensitivity analyses**
To assess the robustness of findings, two additional negative binomial regression models were conducted to examine predictors of repeated incarceration. The first model included participants whose first incarceration occurred before age 18 (n = 82), expanding the sample to account for juvenile-onset cases. The second model was a zero-truncated negative binomial regression restricted to individuals who had been incarcerated at least once, to explore factors associated with the number of incarcerations among this subgroup. Both models used the same covariates as the primary analysis and are presented in Tables B2 and B3.

**Exposure variable**
For negative binomial regression models, an exposure term was included to account for the time each participant was at risk of incarceration. Because these models included individuals first incarcerated as juveniles, we used the age of criminal responsibility in Australia (10 years) as the starting point. Exposure time was calculated by subtracting 10 from the participant’s age at interview (e.g., a participant aged 50 had 40 years of exposure).

**Appendix B: Supplementary results**

**Table B1: Comparison of characteristics between people who were first incarcerated as juveniles and people first incarcerated as adults**

|  | Total sample (%) | First Incarcerated as a juvenile  n (%) | First Incarcerated as adult  n (%) | P value (A vs B) |
| --- | --- | --- | --- | --- |
| N | 255 | 82 | 173 |  |
| Mean age, years (SD) |  |  |  |  |
| Gender |  |  |  | .541 |
| Male | 180 | 58 (70.73) | 122 (70.52) |  |
| Female | 71 | 22 (26.83) | 49 (28.32) |  |
| Highest level of education |  |  |  | .001 |
| Less than 10 years secondary education | 101 | 45 (54.88) | 56 (32.56) |  |
| 10 or more years secondary education | 153 | 37 (45.12) | 116 (67.44) |  |
| Employment status |  |  |  | .520 |
| Employed | 33 | 9 (10.98) | 24 (13.87) |  |
| Unemployed | 222 | 73 (87.06) | 149 (86.13) |  |
| Ever experienced homelessness | 237 | 79 (96.34) | 158 (91.33) | .144 |
| Ever injected drugs | 248 | 81 (98.78) | 167 (96.53) | .305 |
| Hazardous drinking on AUDIT-C*^1^* | 92 | 28 (34.15) | 64 (36.99) | .658 |
| Past year DSM-5 opioid use disorder |  |  |  | .272 |
| Mild | 19 | 9 (13.04) | 10 (6.94) |  |
| Moderate | 17 | 4 (5.80) | 13 (9.03) |  |
| Severe | 177 | 56 (81.16) | 121 (84.03) |  |
| Arrested for opioid use/possession | 123 | 38 (46.91) | 85 (50.30) | .617 |
| Adverse childhood experiences |  |  |  |  |
| Household member used substances | 152 | 48 (76.19) | 104 (70.75) | .419 |
| Mother treated violently | 95 | 31 (50.82) | 64 (44.14) | .380 |
| Physical neglect | 71 | 26 (40.62) | 45 (30.82) | .167 |
| Physical abuse | 119 | 46 (71.88) | 73 (49.32) | .002 |
| Emotional neglect | 114 | 43 (68.25) | 71 (48.30) | .008 |
| Emotional abuse | 132 | 47 (73.44) | 85 (57.43) | .027 |
| Sexual abuse | 102 | 40 (62.50) | 62 (41.61) | .005 |
| Household member had a mental illness | 89 | 26 (41.94) | 63 (43.45) | .840 |
| Parent went to prison | 77 | 27 (42.86) | 50 (34.48) | .250 |
| Parents separated or divorced | 136 | 46 (74.19) | 90 (62.07) | .092 |
| Number of times incarcerated, mean (SD) | 6.8 (8.13) | 11.34 (11.44) | 4.74 (4.84) | <.001 |

**Table B2: Negative binomial regression for whole sample (N = 357)**

|  | **Unadjusted IRR (95% CI)** | **p-value** | **Adjusted IRR (95% CI)** | **p-value** |
| --- | --- | --- | --- | --- |
| Male | 1.98 (1.41, 2.80) | <.001 | 1.86 (1.27, 2.73) | .001 |
| 10 years of secondary schooling or more | 0.41 (0.30, 0.58) | <.001 | 0.47 (0.31, 0.71) | <.001 |
| Age of first illicit drug | 0.96 (0.91, 1.00) | .032 | 1.02 (0.97, 1.07) | .386 |
| <16 (ref) |  |  |  |  |
| > 16 |  |  |  |  |
| Age of first illicit opioid | 0.95 (0.92, 0.97) | <.001 | 0.95 (0.92, 0.98) | .007 |
| <16 (ref) |  |  |  |  |
| > 16 |  |  |  |  |
| Age of first injected illicit drug | 0.96 (0.93, 0.98) | .001 | 0.99 (0.95, 1.03) | .604 |
| <16 (ref) |  |  |  |  |
| > 16 |  |  |  |  |
| Adverse childhood experiences |  |  |  |  |
| Household member used substances | 1.61 (1.12, 2.33) | .011 | 1.33 (0.91, 1.95) | .142 |
| Mother treated violently | 1.31 (0.91, 1.90) | .149 |  |  |
| Physical neglect | 1.62 (1.09, 2.34) | .016 | 1.64 (1.10, 2.44) | .015 |
| Physical abuse | 1.12 (0.77, 1.58) | .583 | -- | -- |
| Emotional abuse | 1.18 (0.82, 1.70) | .381 | -- | -- |
| Emotional neglect | 1.13 (0.78, 1.62) | .519 | -- | -- |
| Sexual abuse | 1.05 (0.73, 1.51) | .778 | -- | -- |
| Household member had a mental illness | 1.17 (0.81, 1.70) | .393 | -- | -- |
| Parent went to prison | 1.47 (0.99, 2.17) | .053 | 1.15 (0.76, 1.76) | .489 |
| Parents separated or divorced | 1.13 (0.78, 1.64) | .509 | -- | -- |
| Constant | -- | -- | 0.22 (0.08, 0.58) | .002 |

**Table B3: Negative binomial regression – zero-truncated model (N =233)**

|  | **Unadjusted IRR (95% CI)** | **p-value** | **Adjusted IRR (95% CI)** | **p-value** |
| --- | --- | --- | --- | --- |
| Male | 1.81 (1.22, 2.70) | .003 | 1.58 (1.01, 2.47) | .044 |
| 10 years of secondary schooling or more | 0.51 (0.36, 0.73) | <.001 | 0.52 (0.34, 0.79) | .002 |
| Age of first illicit drug | 0.98 (0.93, 1.03) | .375 | -- | -- |
| Age of first illicit opioid | 0.95 (0.92, 0.98) | .001 | 0.95 (0.91, 0.99) | .018 |
| Age of first injected illicit drug | 0.97 (0.93, 1.00) | .049 | 1.01 (0.96, 1.06) | .690 |
| Adverse childhood experiences |  |  |  |  |
| Household member used substances | 1.06 (0.67, 1.67) | .806 | -- | -- |
| Mother treated violently | 1.06 (0.70, 1.60) | .781 | -- | -- |
| Physical neglect | 1.43 (0.93, 2.20) | .100 | 1.53 (0.99, 2.36) | .052 |
| Physical abuse | 1.01 (0.67, 1.53) | .945 | -- | -- |
| Emotional abuse | 1.10 (0.72, 1.69) | .641 | -- | -- |
| Emotional neglect | 1.13 (0.75, 1.72) | .556 | -- | -- |
| Sexual abuse | 0.96 (0.63, 1.46) | .848 | -- | -- |
| Household member had a mental illness | 1.09 (0.71, 1.67) | .684 | -- | -- |
| Parent went to prison | 1.06 (0.69, 1.62) | .798 | -- | -- |
| Parents separated or divorced | 0.94 (0.61, 1.44) | .769 | -- | -- |
| Constant | -- | -- | 0.28 (0.11, 0.70) | .007 |

**Table B4: Descriptive statistics of number of times incarcerated**

| **Number of times** | **Frequency** | **%** | **Cumulative %** |
| --- | --- | --- | --- |
| **0** | **124** | **34.73** | **34.73** |
| **1** | **48** | **13.45** | **48.18** |
| **2** | **27** | **7.56** | **55.74** |
| **3** | **30** | **8.40** | **64.15** |
| **4** | **18** | **5.04** | **69.19** |
| **5** | **26** | **7.28** | **76.47** |
| **6** | **15** | **4.20** | **80.67** |
| **7** | **6** | **1.68** | **82.35** |
| **8** | **7** | **1.96** | **84.31** |
| **9** | **2** | **0.56** | **84.87** |
| **10** | **18** | **5.04** | **89.92** |
| **12** | **3** | **0.84** | **90.76** |
| **13** | **1** | **0.28** | **91.04** |
| **15** | **9** | **2.52** | **93.56** |
| **17** | **1** | **0.28** | **93.84** |
| **20** | **11** | **3.08** | **96.92** |
| **25** | **2** | **0.56** | **97.48** |
| **30** | **3** | **0.84** | **98.32** |
| **35** | **3** | **0.84** | **99.16** |
| **40** | **1** | **0.28** | **99.44** |
| **50** | **2** | **0.56** | **100.00** |

**Table B5: Multivariate regression using ACE as a count score**

| **Incarceration** | **Adjusted OR**  **(95% CI)** | **p-value** |
| --- | --- | --- |
| Age category |  |  |
| 18-24 (ref) | -- | -- |
| 25-29 | 0.55 (0.35, 0.85) | 0.007 |
| 30-34 | 0.31 (0.17, 0.56) | <.001 |
| 35+ | 0.17 (0.10, 0.30) | <.001 |
| Male | 1.75 (1.22, 2.51) | 0.002 |
| Education > yr 10 | 0.52 (0.36, 0.76) | 0.001 |
| No drug use (ref) | -- | -- |
| Non-injecting drug use (time-dependent) | 1.50 (0.51, 4.37) | 0.456 |
| Injecting drug use (time-dependent) | 2.86 (1.03, 7.96) | 0.044 |
| ACE score (total) | 1.07 (1.00, 1.14) | 0.034 |
| Constant | .02 | <.001 |
